# The Physical Effects of Child Marriage in India

**Published:** 1892-03

**Authors:** 


### THE PHYSICAL EFFECTS OF CHILD MARRIAGE IN INDIA.

In the *Medical Missionary Record* is published the memorial of fifty-five lady physicians to the Viceroy and Governor-General of India. In their petition the following instances of the results of such marriages have come under the personal observation of one or another of the petitioners:

A. Aged 9. Day after marriage. Left *femur* dislocated, *pelvis* crushed out of shape, flesh hanging in shreds.

B. Aged 10. Unable to stand, bleeding profusely, flesh much lacerated.

C. Aged 9. So completely ravished as to be almost beyond surgical repair. Her husband had two other living wives, and spoke very fine English.

D. Aged 10. A very small child, and entirely undeveloped physically. This child was bleeding to death from the *rectum*. Her husband was a man of about forty years of age, weighing not less than eleven stone (154 lbs.) He had accomplished his desire in an unnatural way.

E. Aged about 9. Lower limbs completely paralyzed.

F. Aged about 12. Laceration of the *perineum* extending through the *sphincter ani*.

G. Aged about 10. Very weak from loss of blood. Stated that great violence had been done her in an unnatural way.

H. Aged about 12. Pregnant, delivered by *craniotomy* with great difficulty, on account of the immature state of the *pelvis* and maternal passage.

I. Aged about 7. Living with husband. Died in great agony after three days.

K. Aged about 10. Condition most pitiable. After one day in hospital was demanded by her husband for his "lawful" use, he said.

L. Aged 11. From great violence done her person, will be a cripple for life. No use of her lower extremities.

M. Aged 10. Crawled to hospital on her hands and knees. Has never been able to stand erect since her marriage.

N. Aged 9. Dislocation of *pubic arch*, and unable to stand or to put one foot before the other.

In view of the above facts, the undersigned lady-doctors and medical practitioners appeal to Your Excellency's compassion to enact or introduce a measure by which the consummation of marriage will not be permitted before the wife has attained the full age of fourteen (14) years. The undersigned venture to trust that the terrible urgency of the matter will be accepted as an excuse for this interruption of Your Excellency's time and attention.

Signed by fifty-five lady-physicians.

---

A Unique Case.—We extract the following from the last issue of the *British Medical Journal*: "A nice point of law has lately been debated before a French Court. The question was whether an operation on a dead body by an unqualified person came within the meaning of the enactment forbidding the illegal practice of medicine. It appears that a pregnant woman had just died, the cause of death not being stated. The curè of the village, who had been with her in her last moments, induced a neighbor who was in the room to perform Cesarean section on the corpse, with a view of saving the child. The operation was successful, but the operator was brought before the magistrate and fined fifteen francs for having been guilty of illegal practice of medicine."

